# Markedly reduced prevalence of *Schistocephalus solidus* in pelagic three-spined sticklebacks (*Gasterosteus aculeatus*) from Lake Constance

**DOI:** 10.1007/s00436-026-08677-3

**Published:** 2026-04-14

**Authors:** Felix Salge, Herwig Stibor, Michael Schubert, Christian Vogelmann

**Affiliations:** 1 Faculty of Chemistry, University of Vienna, Währinger Straße 42, Vienna, A-1090 Austria; 2 Faculty of Biology, Aquatic Ecology, Ludwig-Maximilian University of Munich, Großhaderner Str. 2, Planegg-Martinsried, 82152 Germany; 3 Bavarian State Research Center for Agriculture (LfL). Institute for Fisheries, Weilheimer Str. 8, Starnberg, 82319 Germany

**Keywords:** *Gasterosteus aculeatus*, Invasive species, Lake Constance, *Schistocephalus solidus*, Three-spined stickleback

## Abstract

Host total length, body mass and prevalence of *Schistocephalus solidus* were assessed in pelagic three-spined sticklebacks (*Gasterosteus aculeatus*) from Upper Lake Constance, a prealpine lake in Central Europe, based on 987 individuals collected by pelagic trawl in late autumn 2024. In addition, 62 fish sampled using gillnets in late autumn 2025 were examined using the same biometric and parasitological procedures. The 2024 population was dominated by small-bodied individuals, with a mean total length of 5.26 ± 0.55 cm and a mean body mass of 1.56 ± 0.51 g. Infection was rare: 16 of 987 fish were infected in 2024, corresponding to a prevalence of 1.6%, and parasite index (PI = parasite wet mass / host wet mass) values ranged from 0.013 to 0.302. In 2025, prevalence remained similarly low at 3.2%. Compared with earlier reports from Lake Constance, the late-autumn 2024 sample indicates markedly lower prevalence of *S. solidus* in the pelagic population, and the 2025 sample showed similarly low prevalence.

## Introduction

The ecosystem of Lake Constance, a large prealpine lake in Central Europe, is undergoing significant changes driven by reoligotrophication, i.e. a return toward lower nutrient conditions, which has altered nutrient availability and primary production (Straile and Geller 1998; Ogorelec et al. 2022). These shifts have impacted native fish populations, particularly the economically important whitefish (*Coregonus* spp.), leading to declining fishery yields (Eckmann et al. 2007; Thomas et al. 2010; Roch et al. 2018).

Coinciding with these changes, the invasive three-spined stickleback (*Gasterosteus aculeatus*), first recorded in the 1950s, has established a large pelagic population since 2012, becoming a dominant species in the open water zone (Alexander et al. 2016; Roch et al. 2018; Eckmann and Engesser 2019). This pelagic expansion has raised concerns about its ecological impact, particularly regarding resource competition with and predation on native planktivorous fish (Hudson et al. 2021; Bretzel et al. 2021). *Schistocephalus solidus* is a common cestode parasite of three-spined sticklebacks, and the stickleback–*S. solidus* system is an established model for investigating host–parasite interactions in fish because infection can affect host growth, reproductive development and behaviour (Barber and Scharsack 2010). The parasite has a three-host life cycle involving cyclopoid copepods as first intermediate hosts, three-spined sticklebacks as specific second intermediate hosts, and mainly fish-eating birds as definitive hosts (Barber and Scharsack 2010). Recent work has further underscored the evolutionary relevance of the stickleback–*S. solidus* system for understanding host responses to parasitism (Weber et al. 2022). In Lake Constance, previous studies reported substantially higher prevalence in pelagic sticklebacks during the recent period of high pelagic abundance, as well as pronounced seasonal and interannual variation in infection levels (Baer et al. 2022; Bretzel et al. 2021). Following this recent period of high pelagic stickleback abundance, updated information on the parasitological status of pelagic sticklebacks remains limited. Changes in host density and habitat use may influence parasite transmission and, consequently, infection prevalence. Here, we quantify host total length, body mass and infection with the cestode *S. solidus* in a large late-autumn sample of pelagic three-spined sticklebacks collected in 2024, complemented by additional observations from 2025. This study provides an updated snapshot of host–parasite dynamics in a population potentially transitioning from a period of high pelagic stickleback abundance to lower population density.

## Materials and methods

The sampling was conducted over three consecutive nights (early November 2024) using several pelagic trawl hauls in the upper basin of Lake Constance (Bodensee-Obersee) at a depth of 4–9 m. Late-autumn night sampling was chosen because pelagic sticklebacks are readily accessible to targeted trawling in the upper water column during this period, with minimal bycatch. In total, approximately 3000 sticklebacks were caught, from which 987 individuals were randomly selected for detailed biometric and parasitological analyses. The trawl net used had a 125 mm mesh size at the opening and a 6 mm mesh size in the codend. Immediately following capture, fish were euthanized using a licensed electric device and frozen for subsequent laboratory examination. In the laboratory, the 987 specimens were thawed for processing. For the biometric analysis, the total length (TL) of each fish was measured to the nearest millimetre using a measuring board, and the total body weight was recorded to the nearest 0.1 gram using a precision balance. Sex and age were not determined. Subsequently, each fish was dissected and examined for the presence of the cestode parasite *S. solidus*.

Pelagic sticklebacks were also sampled in late autumn 2025 using pelagic multimesh gillnets in the same lake basin. A total of 62 individuals were analysed using identical biometric and parasitological procedures. Because the 2025 sample was collected using different gear and yielded only two infected fish, these data were not used for formal year-to-year comparisons. However, the two infected 2025 fish were included in the pooled exploratory analysis of parasite index versus host total length shown in Fig. 2. Parasitological terminology followed Bush et al. (1997). Parasite prevalence was defined as the proportion of hosts infected with one or more individuals of *S. solidus*. Mean intensity was defined as the mean number of parasites per infected host. Parasite wet mass was recorded for infected individuals, and the parasite-host biomass ratio (PI) was calculated following Pennycuick (1971) as parasite wet mass relative to host wet mass. Because only 18 infected individuals were available across both sampling years, the relationship between parasite index and host total length was explored using simple linear regression on pooled infected fish from 2024 to 2025. All statistical analyses were performed in R version 4.2.3 (R Core Team 2023).

## Results and discussion

**Fig. 1:**
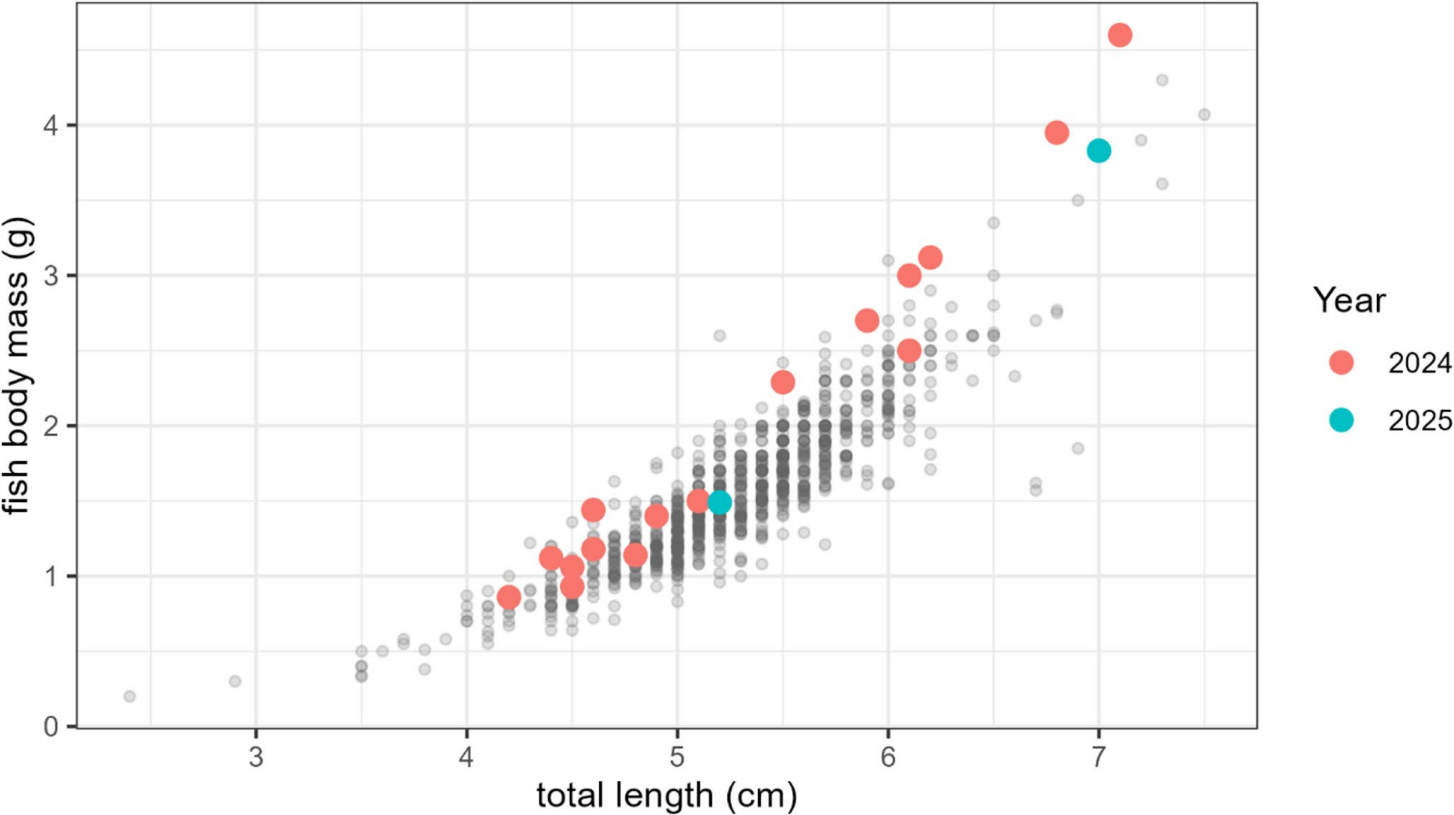
Relationship between total length (cm) and body mass (g) of pelagic three-spined sticklebacks sampled in Lake Constance in late autumn 2024 and 2025. Grey points represent uninfected individuals (2024: *n* = 971; 2025: *n* = 60). Coloured points indicate individuals infected with Schistocephalus solidus (2024: *n* = 16; 2025: *n* = 2)

The pelagic stickleback population sampled in late autumn 2024 consisted predominantly of small-bodied individuals (Table 1; Fig. 1). Mean total length was 5.26 ± 0.55 cm (range: 2.4–7.3 cm), and mean body mass was 1.56 ± 0.51 g (range: 0.20–4.60 g).

**Table 1:** Biometric traits and infection parameters of pelagic three-spined sticklebacks (*Gasterosteus aculeatus*) sampled in Lake Constance in late autumn 2024 and 2025. Parasite index (PI) represents parasite wet mass relative to host wet mass

Year	*N* analysed	Total length (cm) mean ± SD (range)	Body mass (g) mean ± SD (range)	Parasite prevalence (%)	Parasite index (PI, range)
2024	987	5.26 ± 0.55 (2.4–7.3)	1.56 ± 0.51 (0.20–4.60)	1.6	0.013–0.302
2025	62	5.17 ± 0.52 (4.3–7.5)	1.40 ± 0.56 (0.81–4.07)	3.2	0.063–0.128

Infection with *S. solidus* was rare. Only 16 of 987 individuals were infected, corresponding to a parasite prevalence of 1.6% (Table 1). A total of 18 parasites were recorded, resulting in a mean infection intensity of 1.13 parasites per infected host. Parasite index (PI) values ranged from 0.013 to 0.302, corresponding to parasite wet masses between 0.02 and 0.76 g in infected individuals. Most infected individuals (11 of 16; 68.8%) had a total length below 6 cm.

**Fig. 2:**
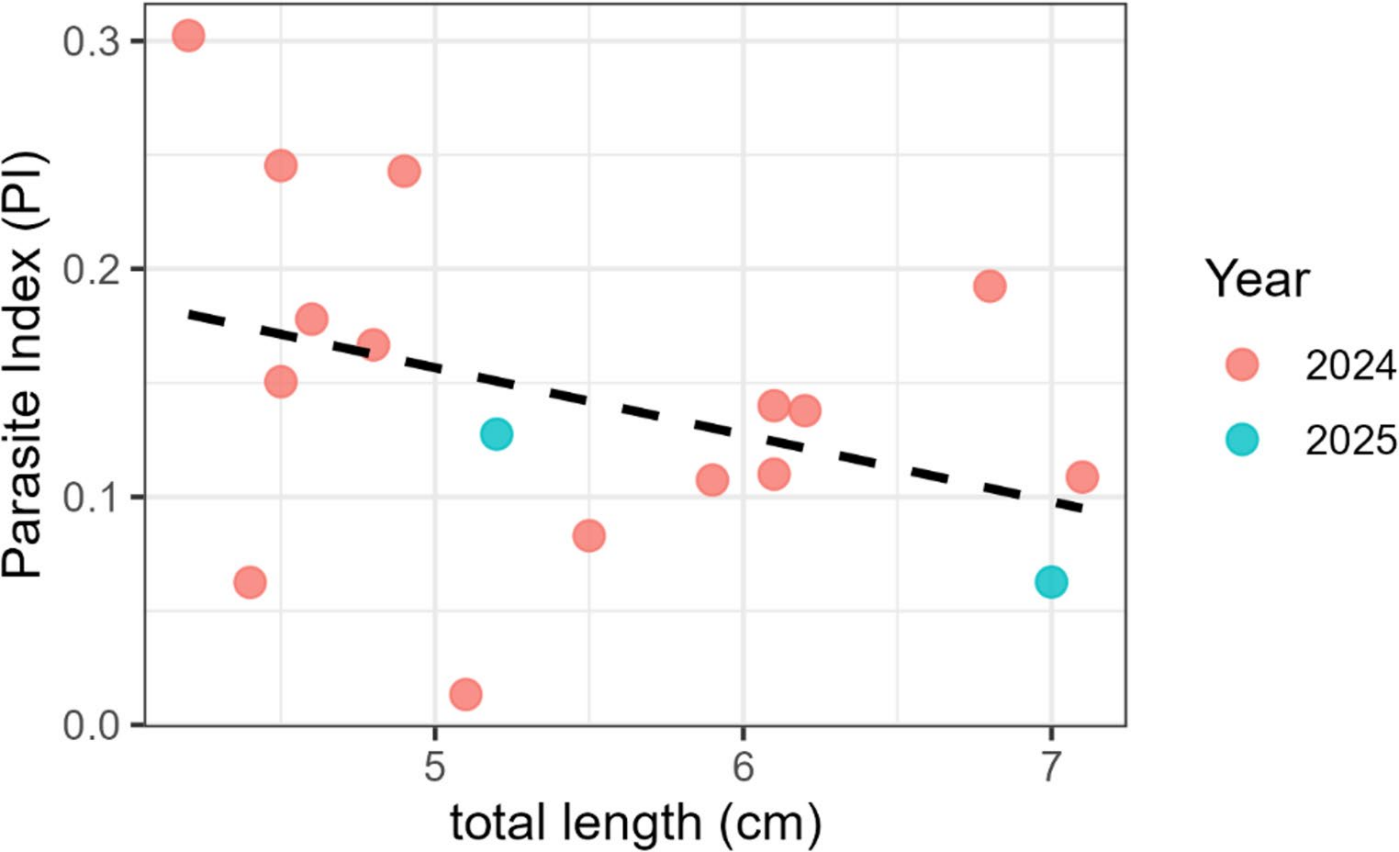
Relationship between parasite index (PI) and total length in infected three-spined sticklebacks sampled in late autumn 2024 and 2025. Each point represents one infected individual. The dashed line indicates the fitted linear trend across pooled infected fish from both sampling years (2024 and 2025)

In late autumn 2025, parasite prevalence was similarly low (3.2%; 2 of 62 individuals infected), with PI values ranging from 0.063 to 0.128, corresponding to parasite wet masses between 0.19 and 0.24 g (Table 1). These observations are consistent with the low prevalence observed in 2024, although the 2025 sample was small and collected with different gear. When infected individuals from both sampling years were analysed together (*n* = 18), parasite index showed a weak negative trend with increasing host total length (Fig. 2), but the relationship was not statistically significant (linear regression: slope = − 0.029, 95% CI: −0.069 to 0.010, *p* = 0.13).

The pelagic stickleback population sampled in late autumn 2024 was characterized by a low prevalence of infection with *S. solidus*. With only 1.6% of individuals infected, parasite prevalence was markedly lower than values reported during earlier years of high pelagic stickleback abundance in Lake Constance, including mean prevalences of 23% for 2016–2019 (Baer et al. 2022) and 13.8% in autumn 2018 (Bretzel et al. 2021). Similarly low prevalence was observed in the 2025 sample (3.2%), although this sample was smaller and collected using different gear. These differences are consistent with previous observations of strong seasonal and interannual fluctuations in parasite infection levels of pelagic stickleback populations in Lake Constance (Baer et al. 2022).

Although the sampled population was dominated by small-bodied individuals, host age was not determined and the observed size structure therefore cannot be assigned unambiguously to specific age classes. Seasonal and parasite-mediated mortality may nonetheless contribute to late-autumn size distributions if larger, heavily infected individuals are selectively lost from the population (Baer et al. 2022).

Low prevalence in late autumn may result from several non-exclusive mechanisms. Because *S. solidus* has a complex trophic life cycle involving cyclopoid copepods, sticklebacks and mainly fish-eating birds, temporal variation in the abundance or spatial overlap of these hosts could influence transmission (Barber and Scharsack 2010). Seasonal shifts in habitat use between pelagic and littoral zones may also alter exposure to infected copepods (Baer et al. 2024). Because these drivers were not quantified here, the present study does not identify the mechanism underlying the observed low prevalence, but documents a low-prevalence snapshot of the pelagic population.

Beyond these mechanisms, density-dependent processes may also contribute to temporal variation in parasite prevalence. Lower host densities can reduce transmission probabilities and may also alleviate intraspecific resource competition, both of which could contribute to reduced infection levels. This is particularly relevant for pelagic stickleback populations in Lake Constance, where lake-wide surveys have documented periods of extreme pelagic dominance, characterized by very high numerical abundance and biomass relative to other fish species (Eckmann and Engesser 2019). Density-dependent variation in parasite prevalence has been demonstrated across a range of host–parasite systems, where reduced host densities were associated with lower infection levels, providing broader ecological support for this conceptual framework (Hasik et al. 2025).

Pelagic stickleback populations in Lake Constance are known to undergo pronounced fluctuations in abundance and biological condition (Numann 1972; Baer et al. 2022, 2024; Ogorelec et al. 2022). Periods of high pelagic abundance have previously been associated with increased parasite pressure and ecological interactions with native planktivorous fishes (Roch et al. 2018; Hudson et al. 2021; Bretzel et al. 2021). In this context, the present findings document a pelagic population with low parasite prevalence following a period of high abundance. Whether this pattern reflects a transient post-peak state, or a longer-lasting shift cannot be resolved from the present snapshot.

Because sampling was restricted to late autumn and sex and age were not determined, the present data should be interpreted as a temporally restricted snapshot rather than a full population assessment. Snapshot resampling can be informative when long-term parasitological monitoring is limited, but its low temporal resolution constrains inference about directional long-term change (Hammoud et al. 2025). Repeated sampling across seasons and years will therefore be necessary to determine whether the present low-prevalence pattern persists.

## Data Availability

The datasets generated and/or analysed during the current study are available from the corresponding author on reasonable request.

## References

[CR1] Alexander TJ, Vonlanthen P, Seehausen O et al (2016) Artenvielfalt und Zusammensetzung der Fischpopulation im Bodensee. Projet Lac, Eawag, Kastanienbaum. https://www.ibkf.org/wpcontent/uploads/2018/03/ProjetLac_Bodensee_2014_fin_web.pdf. Accessed 10 March 2026

[CR2] Baer J, Gugele SM, Roch S, Brinker A (2022) Stickleback mass occurrence driven by spatially uneven parasite pressure? Insights into infection dynamics, host mortality, and epizootic variability. Parasitol Res 121:1607–1619. 10.1007/s00436-022-07517-4 35435510 10.1007/s00436-022-07517-4 PMC9098546

[CR3] Baer J, Ziegaus S, Schumann M et al (2024) Escaping malnutrition by shifting habitats: A driver of three-spined stickleback invasion in Lake Constance. J Fish Biol 104:746–757. 10.1111/jfb.15622 37984830 10.1111/jfb.15622

[CR4] Barber I, Scharsack JP (2010) The three-spined stickleback-Schistocephalus solidus system: an experimental model for investigating host-parasite interactions in fish. Parasitology 137:411–424. 10.1017/S0031182009991466 19835650 10.1017/S0031182009991466

[CR5] Bretzel JB, Geist J, Gugele SM et al (2021) Feeding ecology of invasive three-spined stickleback (Gasterosteus aculeatus) in relation to native juvenile Eurasian perch (Perca fluviatilis) in the pelagic zone of Upper Lake Constance. Front Environ Sci 9:670125. 10.3389/fenvs.2021.670125

[CR6] Bush AO, Lafferty KD, Lotz JM, Shostak AW (1997) Parasitology meets ecology on its own terms: Margolis. Revisit J Parasitol 83:575–583. 10.2307/3284227 9267395

[CR7] Eckmann R, Engesser B (2019) Reconstructing the build-up of a pelagic stickleback (Gasterosteus aculeatus) population using hydroacoustics. Fish Res 210:189–192. 10.1016/j.fishres.2018.08.002

[CR8] Eckmann R, Kugler M, Ruhlé C (2007) Evaluating the success of large-scale whitefish stocking at Lake Constance. Adv Limnol 60:361–368

[CR9] Hammoud C, Balbuena JA, Blasco-Costa I, O’Dwyer K, Paterson RA, Scholz T, Selbach C, Sures B, Thieltges DW (2025) Long-term trends in parasite diversity and infection levels: approaches and patterns. Biol Rev. 10.1002/brv.70119 41416625 10.1002/brv.70119 PMC13149780

[CR10] Hasik AZ, Butt S, Maris K et al (2025) Population density drives increased parasitism via greater exposure and reduced resource availability in wild red deer. Parasitology 152:724–734. 10.1017/S0031182025100516 40659043 10.1017/S0031182025100516 PMC12418284

[CR11] Hudson CM, Lucek K, Marques DA et al (2021) Threespine stickleback in Lake Constance: The ecology and genomic substrate of a recent invasion. Front Ecol Evol 8:611672. 10.3389/fevo.2020.611672

[CR12] Numann W (1972) The Bodensee: Effects of exploitation and eutrophication on the salmonid community. J Fish Res Board Can 29:833–847. 10.1139/f72-127

[CR13] Ogorelec Ž, Brinker A, Straile D (2022) Small but voracious: invasive generalist consumes more zooplankton in winter than native planktivore. NeoBiota 78:71–97. 10.3897/neobiota.78.86788

[CR14] Pennycuick L (1971) Seasonal variations in the parasite infections in a population of three-spined sticklebacks, Gasterosteus aculeatus L. Parasitology 63:373–388. 10.1017/S0031182000079919 5139023 10.1017/s0031182000079919

[CR15] R Core Team (2023) R: A Language and Environment for Statistical Computing. R Foundation for Statistical Computing, Vienna, Austria

[CR16] Roch S, Von Ammon L, Geist J, Brinker A (2018) Foraging habits of invasive three-spined sticklebacks (Gasterosteus aculeatus) – impacts on fisheries yield in Upper Lake Constance. Fish Res 204:172–180. 10.1016/j.fishres.2018.02.014

[CR17] Straile D, Geller W (1998) Crustacean zooplankton in Lake Constance from 1920 to 1995: Response to eutrophication and re-oligotrophication. Adv Limnol 53:255–274

[CR18] Thomas G, Rösch R, Eckmann R (2010) Seasonal and long-term changes in fishing depth of Lake Constance whitefish. Fish Manag Ecol 17:386–393. 10.1111/j.1365-2400.2010.00734.x

[CR19] Weber JN, Steinel NC, Peng F et al (2022) Evolutionary gain and loss of a pathological immune response to parasitism. Science 377(6611):1206–1211. 10.1126/science.abo3411 36074841 10.1126/science.abo3411 PMC9869647

